# Supplementary material for: Alzheimer-mutant γ-secretase complexes stall amyloid β-peptide production
Source: bioRxiv. 2024 Aug 31:2024.08.30.610520. Preprint. [Version 1] doi: 10.1101/2024.08.30.610520 (PMC11383658; doi:10.1101/2024.08.30.610520)
Supplement: Supplementary file 1 [file NIHPP2024.08.30.610520v1-supplement-1.pdf]

bioRxiv preprint doi: https://doi.org/10.1101/2024.08.30.610520; this version posted August 31, 2024. The copyright holder for this preprint (which was not certified by peer review) is the author/funder, who has granted bioRxiv a license to display the preprint in perpetuity. It is made available under aCC-BY 4.0 International license.

SUPPLEMENTAL INFORMATION

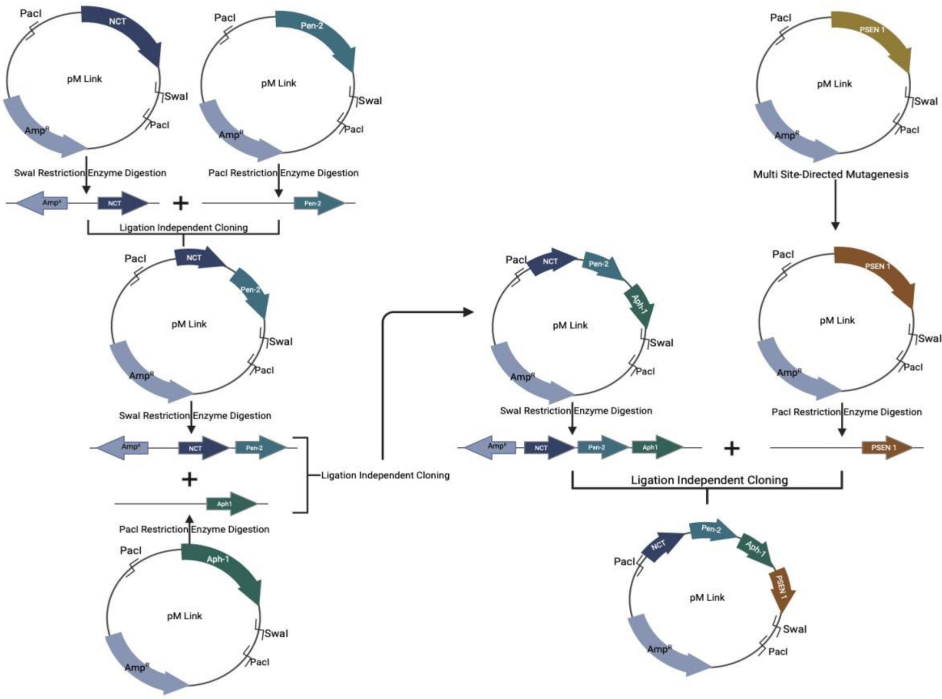

Figure S1. Process of installing PSEN-1 mutations into full γ-secretase plasmid. Step-by-step Ligation-Independent Cloning (LIC) was developed in *E. coli*, along with restriction digestion of both the insert and vector, enabling the successful insertion of mutations. A tricistronic plasmid containing genetic codes for three membrane protein components of the γ-secretase complex, including nicastrin, presenilin enhancer (Pen2), and anterior pharynx-defective 1 (Aph1) was prepared. This plasmid was created in two steps: initially, Nicastrin and Pen2 were combined using LIC in *E. coli* and restriction digestion, forming a bicistronic plasmid. Subsequently, the bicistronic plasmid was further modified by including Aph1 through another round of restriction digestion and LIC in *E. coli*, resulting in a tricistronic plasmid. Finally, Multi-Site Directed Mutagenesis was used to mutate PSEN1, and this monocistronic construct was incorporated into the tricistronic plasmid through additional rounds of restriction digestion and LIC in *E. coli*. Figure 1 illustrates the details of this process.

bioRxiv preprint doi: https://doi.org/10.1101/2024.08.30.610520; this version posted August 31, 2024. The copyright holder for this preprint (which was not certified by peer review) is the author/funder, who has granted bioRxiv a license to display the preprint in perpetuity. It is made available under aCC-BY 4.0 International license.

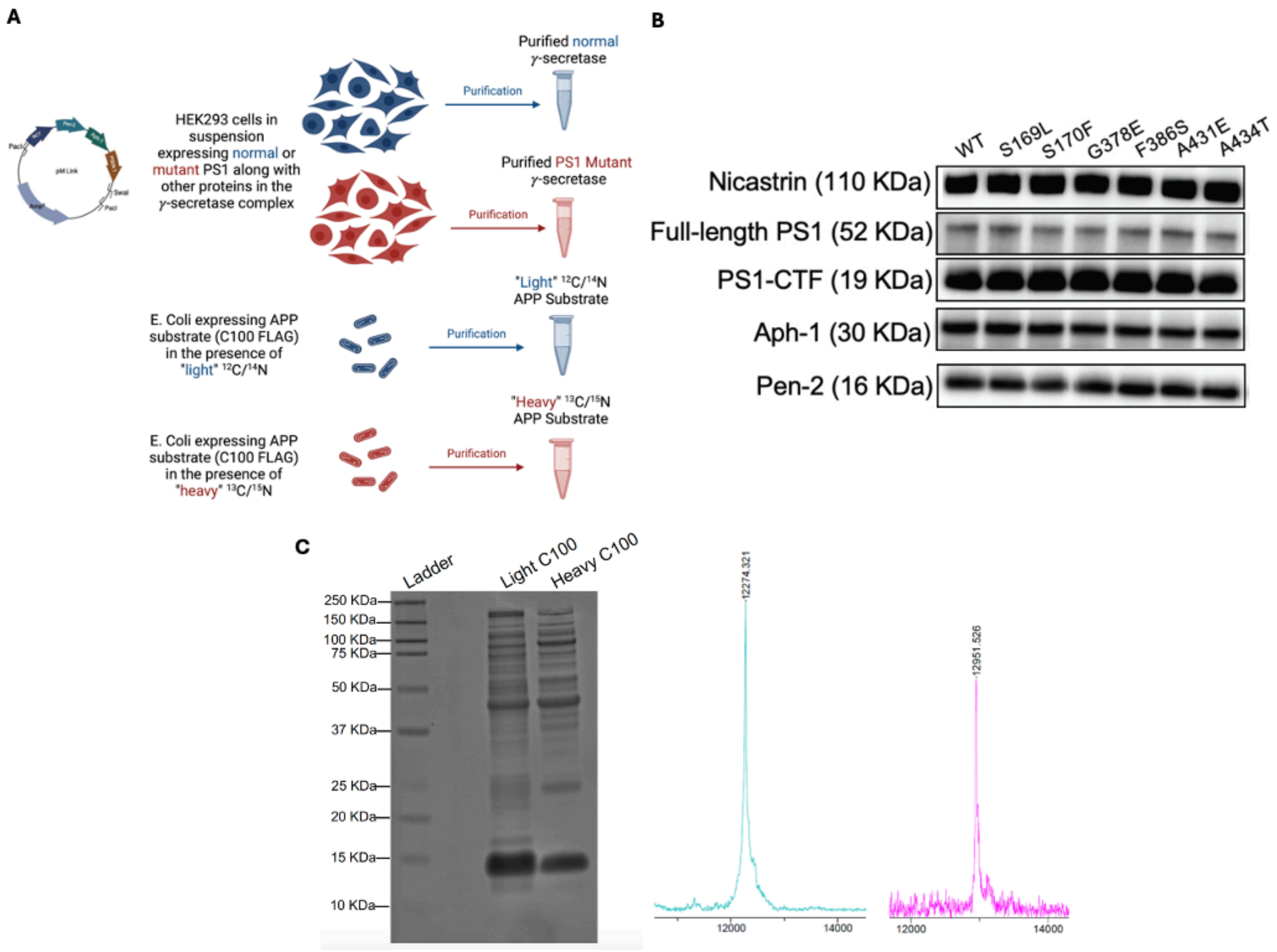

Figure S2. Expression, purification, and quality control of C100 substrate and γ-secretase. (A) Schematic of expression and purification of γ-secretase and C100. (B) Western blot analysis of all components within expressed and purified WT and FAD-mutant γ-secretase complexes, normalized to protein concentration using Pen2 intensity. (C) Characterization of light and heavy isotopic C100-FLAG substrates. The identity and purity of both C100-FLAG variants were assessed using SDS-PAGE with silver staining and MALDI-TOF mass spectrometry. The theoretical masses are 12272.89 for the light C100-FLAG and 12951.6 for the heavy C100-FLAG. Prior to reactions, concentrations were normalized based on band intensity in western blotting.

bioRxiv preprint doi: https://doi.org/10.1101/2024.08.30.610520; this version posted August 31, 2024. The copyright holder for this preprint (which was not certified by peer review) is the author/funder, who has granted bioRxiv a license to display the preprint in perpetuity. It is made available under aCC-BY 4.0 International license.

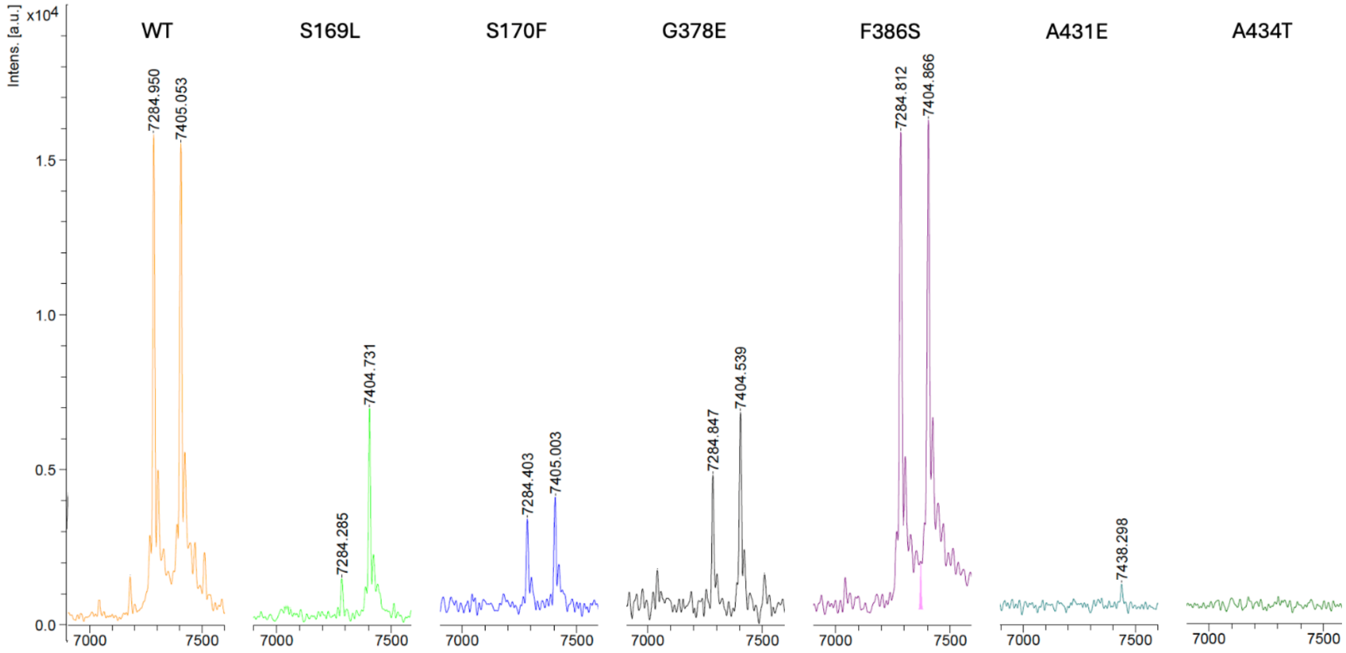

Figure S3. MALDI-TOF MS detection of AICD 50-99 and AICD 49-99 products from wild-type (WT) and six PSEN1 FAD-mutant γ-secretase. The theoretical mass for AICD 50-99 is 7286.02 Da, and for AICD 49-99 is 7406.12 Da.

bioRxiv preprint doi: https://doi.org/10.1101/2024.08.30.610520; this version posted August 31, 2024. The copyright holder for this preprint (which was not certified by peer review) is the author/funder, who has granted bioRxiv a license to display the preprint in perpetuity. It is made available under aCC-BY 4.0 International license.

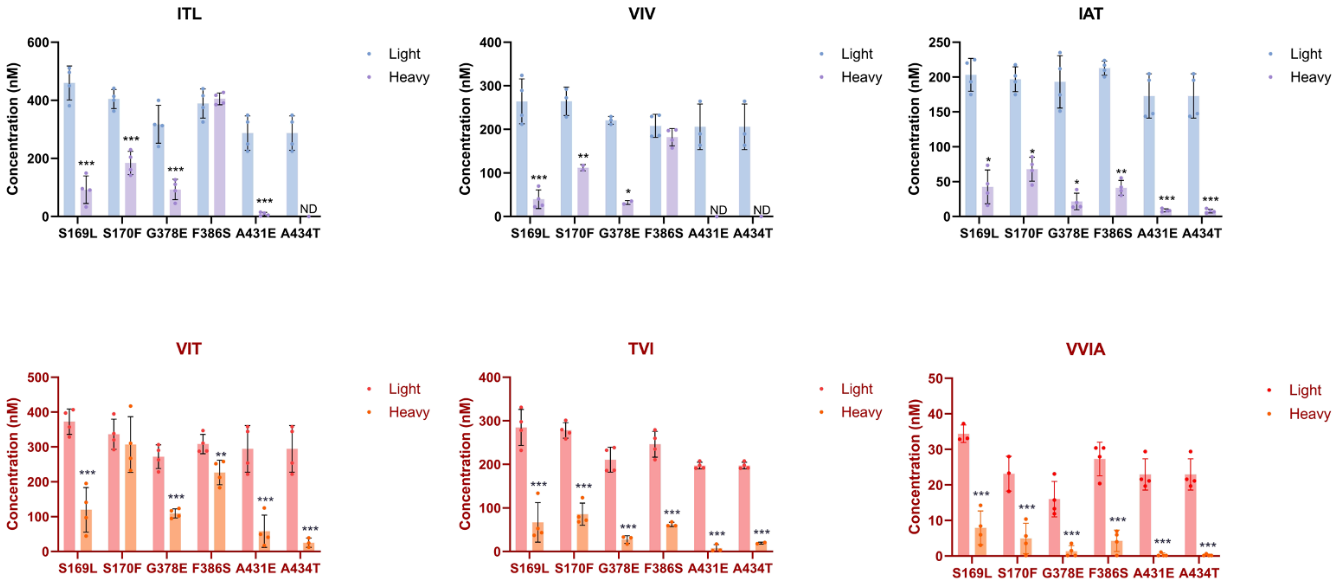

Figure S4. Alzheimer-mutant PSEN-1 affects the processive proteolysis of C99 by γ-secretase. Bar graphs illustrating coproduct formation at each trimming step. For the Aβ49/Aβ40 pathway, blue and purple bars represent the first, second, and third trimming steps. Red and orange bars denote trimming steps for the Aβ48/Aβ38 pathway. Blue/red and Purple/orange bars indicate coproducts formed by WT and FAD-mutant γ-secretase, respectively. For each graph, $n = 4$ and statistical significance was determined using unpaired two-tailed t-tests comparing FAD mutants with WT (*$p < 0.05$, **$p < 0.01$, ***$p < 0.001$). (Note: This figure is another representation of Figure 2C).

bioRxiv preprint doi: https://doi.org/10.1101/2024.08.30.610520; this version posted August 31, 2024. The copyright holder for this preprint (which was not certified by peer review) is the author/funder, who has granted bioRxiv a license to display the preprint in perpetuity. It is made available under aCC-BY 4.0 International license.

Table S1. Cross Reactivity of Aβ43 peptide with Aβ40 and Aβ42 ELISA kits.

A

Concentration of Aβ43 (pg/mL)	Cross reactivity (Read: pg/mL)
**15.63**	8.9
**31.25**	10.5
**62.5**	12.0
**125**	14.2
**250**	28.8
**500**	67.4
**1000**	203.3
**2500**	867.4
**5000**	OF
**10000**	OF
**20000**	OF
**200000**	OF
**1000000**	OF

B

Concentration of Aβ43 (pg/mL)	Cross reactivity (Read: pg/mL)
**7.8**	15.8
**15.63**	11.4
**31.25**	22.6
**62.5**	7.1
**125**	15.0
**250**	20.7
**500**	32.5
**1000**	41.6
**50000**	93.4
**100000**	104.2
**200000**	116.3
**1000000**	117.4

A. Cross-reactivity of Aβ43 with Aβ42 in ELISA assays. Various concentrations of Aβ43 (ranging from 15.63 pg/ml to 1000000 pg/ml) were tested using Aβ42-specific ELISA kits. The instrument readings for each concentration are displayed, indicating significant cross-reactivity starting at 250 pg/ml (0.06 nM) of Aβ43. (Note: "OF" stands for overflow)
B. Cross-reactivity of Aβ43 with Aβ40 in ELISA assays. Different concentrations of Aβ43 (ranging from 7.8 to 1,000,000 pg/ml) were assessed using ELISA kits specific for Aβ40. The resulting instrument readings for each concentration are presented, revealing cross-reactivity beginning at 500 pg/ml (0.12 nM) of Aβ43.
